# *In Vivo* Healing Potential of *Aegle marmelos* in Excision, Incision, and Dead Space Wound Models

**DOI:** 10.1155/2014/740107

**Published:** 2014-03-04

**Authors:** M. K. Gautam, V. Purohit, M. Agarwal, A. Singh, R. K. Goel

**Affiliations:** Department of Pharmacology, Faculty of Modern Medicine, Institute of Medical Sciences, Banaras Hindu University, Varanasi 221005, India

## Abstract

The study incorporates the wound healing potential of *Aegle marmelos* fruit pulp extract (AME) on excision, incision, and dead space wound models in rats. AME (200 mg/kg) was administered orally once daily for variable days depending on the type of wound ulcer study. AME was studied for its wound breaking strength (incision wound), rate of contraction, period of epithelization and histology of skin (excision model), and granulation tissue free radicals, antioxidants, acute inflammatory marker, and connective tissue markers and deep connective tissue histology (dead space wound). Complete wound contraction and epithelization were observed at the 20th day after treatment with AME as compared to the 24th day in control rats. Mean epithelization period and scar area were decreased while wound breaking strength was increased with AME compared with control. Granulation tissue showed increased levels of collagen determinants (33.7 to 64.4%, *P* < 0.001) and antioxidants (13.0 to 38.8%, *P* < 0.05 to *P* < 0.001), whereas markers of oxidative stress (55.0 to 55.6%, *P* < 0.001) and myeloperoxidase (21.3%, *P* < 0.001) were decreased in AME treated group. *A. marmelos* seems to promote wound healing by enhancing connective tissue formation and antioxidants status with decrease in free radicals and myeloperoxidase having tissue damaging effects.

## 1. Introduction

Wounds are still a major problem in developing countries, often having severe complications and involving high costs for therapy. Healing is a complex process that involves a series of biochemical and cellular reactions initiated in response to an injury that restores the function and integrity of damaged tissues. Repair of injured tissues occurs as a sequence of events, which includes inflammation, proliferation, and migration of different cell types [1]. It is consented that reactive oxygen species (ROS) are deleterious to wound healing process due to the harmful effects on cells and tissues [2]. Free radical scavenging enzymes (FRSE) are a cytoprotective enzymal group that has an essential role in the reduction, deactivation, and removal of ROS, as well as regulating the wound healing process. Wound related non-phagocytic cells also generate free radicals by involving non-phagocytic oxidase mechanism [3]. Imbalance in free radical generations and antioxidants caused delayed wound healing. Therefore, elimination of ROS could be an important factor in wound healing [4].

*Aegle marmelos* (family: Rutaceae, AM) known as bael in Hindi and grows up to 18 meters tall and bears thorns and fragrant flowers. AM tree is indigenous to dry forests on hills and plains of India, Pakistan, Bangladesh, Sri Lanka, Myanmar, Nepal, Vietnam, Laos, Cambodia, and Thailand. It has a woody-skinned, smooth fruit 5–15 cm in diameter. It has numerous seeds, which are densely covered with fibrous hairs and are embedded in a thick, gluey, aromatic pulp. According to Indian traditional system, it been used to treat pain, fever, inflammation, respiratory disorders, cardiac disorders, dysentery, and diarrhoea. AM have evaluated many pharmacological activities such as anti-inflammatory, antipyretic and analgesic, antidiarrheal, antidiabetic, antifungal, antihyperlipidemic, antimicrobial, antibacterial and antiparasitic, anticancer, antimalaria, hepatoproctective, anticolitis, and cardioprotective activities [5, 6]. The fruit is reported to contain many functional and bioactive compounds such as carotenoids, phenolics, alkaloids, coumarins, flavonoids, terpenoids, and other antioxidants [7].

Since ancient times, people have used plants and preparations thereof to accelerate the wound healing process. Herbal medicines in wound management involve disinfection, debridement and the provision of suitable environment for natural healing process [8]. In fact, alternative medicine is of less toxicity and with fewer side effects compared with conventional medicine, and hence it is important to introduce a scientific validation for the medicinal effect of plants used in traditional medicine. In this context, the present study was therefore, undertaken to evaluate the wound healing potential of 50% ethanol extract of *Aegle marmelos *fruit pulp in incision, excision and dead space wound models in rats.

## 2. Materials and Methods

### 2.1. Animals

Inbred Charles-Foster albino rats (180–230 g) of either sex were obtained from the central animal house of Institute of Medical Sciences, Banaras Hindu University, Varanasi. They were kept in the departmental animal house at 26 ± 2°C and relative humidity 44–56%, light and dark cycles of 10 and 14 h, respectively, for 1 week before and during the experiments. Animals were provided with standard rodent pellet diet and water *ad libitum*. “Principles of laboratory animal care” (NIH publication no. 82-23, revised 1985) guidelines were followed. Approval from the Institutional Animal Ethical Committee was taken prior to the experimental work (Notification no. - Dean/2010-11/173 dated 23.07.2010).

### 2.2. Plant Material

Big sized, unripe, AM fruits were collected during months of November to March. The shell of the fruit was removed and the pulp was cut into small pieces and dried at room temperature and powdered and stored for further use. The plants and their parts were identified with the standard sample preserved in the Department of Dravyaguna, Institute of Medical Sciences, Varanasi.

### 2.3. Preparation of Extracts

50% ethanol extract of AM (AME) was prepared by adding 1 liter of ethanol and water (1 : 1) in 200 g of dried fine powder of AM. The mixture was shaken at intervals and the extract so obtained was filtered after an interval of two days. The procedure was repeated twice at an interval of two days. The extract so obtained each time was mixed and later dried at 40°C in incubator. The yield of the extracts was 12.7%. Enough quantity of the extract was prepared fresh before use.

### 2.4. Drugs and Chemicals

Vitamin E (VTE), ethylene diamine tetra acetic acid (EDTA), pyridine, vanadium chloride, and sodium nitrite were purchased from Merck Ltd., Mumbai, India, and thiobarbituric acid (TBA), 1,1,3,3-tetramethoxypropane, Griess reagent, Tris buffer, sodium pyrophosphate, phenazine methosulphate (PMS), nitroblue Tetrazolium (NBT), nicotineamide adenine dinucleotide (NADH), and hexadecyltrimethylammonium bromide (HTAB) were purchased from Sigma-Aldrich, Co., St. Louis, MO, USA. Dithiobis nitro benzoic acid (DTNB) were purchase from Hi Media Laboratory, Mumbai, India. All of the other chemicals were used of analytical grade.

### 2.5. Dose Selection and Treatment Protocol

A preliminary dose-response effect using AME was undertaken to study the wound breaking strength in incision wound model in rat. Graded doses of AME 100, 200, and 400 mg/kg were administered once daily by orogastric tube for 10 days in rats following induction of incision wound. The sutures were removed on the 7th day of the experiment and wound breaking strength (WBS) in g was measured on 10th post-wounding day. AME 200 mg/kg dose showed optimal effect in incision wound model therefore; it was chosen for further study on excision and dead space wound models (Table 1).

AME and VTE (200 mg/kg) suspended in 0.5% carboxy methyl cellulose (CMC) in distilled water were given orally once daily from day 1, 4 hour after the induction of excision and dead space wound models. The animals received AME/VTE, orally with the help of an orogastric tube in the volume of 10 mL/kg body weight. AME was administered once daily for 10 days for dead space wound study and for 20 days or till the period of complete epithelization in excision wound study while control rats received 0.5% CMC for the same period in respective studies.

### 2.6. Wound Healing Studies

#### 2.6.1. Incision Wound Model

Two parallel 6 cm paravertebral incisions were made through the full thickness of the skin, 1 cm lateral to the midline of vertebral column after giving anaesthesia [1]. Wounds were closed with interrupted sutures, 1 cm apart, with surgical suture. The sutures were removed on the 7th postwounding day. Wound breaking strength (WBS) was measured on the 10th postwounding day in anaesthetized rats. A line was drawn on either side of the incision line 3 mm away from the wound. Two Allis forceps were firmly applied on to the line facing each other. One of the forceps was fixed, while the other was connected to a freely suspended lightweight polypropylene graduated container through a string run over to a pulley. Standard weights were put slowly and steadily into the container. A gradual increase in weight was transmitted to the wound site pulling apart the wound edges. As the wound just opened up, the weight was stopped and noted.

#### 2.6.2. Excision Wound Model

Rats were anesthetized with ketamine hydrochloride (50 mg/kg), an area of about *≈*500 mm^2^ was marked on the back of the rat by a standard ring, and then full thickness of the marked skin was cut carefully. Wounds were traced on 1 mm^2^ graph paper on the day of wounding and subsequently at a gap period of 4 days till 12th day, then on the alternate days until healing was complete. Changes in wound area were measured regularly and the rate of wound contraction calculated as given in the formula below.

(1) $$\begin{array}{c} \text{Percent}\;\text{wound}\;\text{contraction}=\frac{\text{healed}\;\text{area}}{\text{total}\;\text{wound}\;\text{area}}\times 100. \end{array}$$

Significance in wound healing of the test groups are derived by comparing healed wound area on respective days with healed wound area of control group. The period of epithelization that is, day of fall of eschar and scar area were also noted down [1]. where, healed area = original wound area − present wound area.

#### 2.6.3. Dead Space Wound Model

Rats were anesthetized by ketamine hydrochloride and 1 cm incision was made on dorso-lumbar part of the back. Two polypropylene tubes (0.5 × 2.5 cm^2^ each) were placed in the dead space of lumbar region of rat on each side, and wounds were closed with a suture material. On the 10th postwounding day, the animals were sacrificed and granulation tissue formed on and around the implanted tubes was carefully dissected out, weighed, and processed for the estimation of free radicals, antioxidants, and collagen tissue parameters [4].

### 2.7. Estimation of Granulation Tissue Antioxidants in Wet Tissue

#### 2.7.1. Estimation of Glutathione (GSH)

1 mL of tissue homogenate (100 mg/mL in phosphate buffer) was mixed in 15 mL test tube with 0.8 mL of distilled water and 0.2 mL of 50% TCA. The tubes were shaken intermittently for 10–15 min and centrifuged for 15 min at 3000 rpm for 10 min. 0.6 mL of supernatant was mixed with 0.8 mL of 0.4 M Tris buffer (pH 8.9) and 20 µL of 0.1 M DTNB in absolute methanol, and the sample was shaken. The absorbance was read within 5 min of the addition of 40 µL DTNB at 412 nm against a reagent blank with no homogenate. The results were expressed as nM/mg protein [9].

#### 2.7.2. Estimation of Superoxide Dismutase (SOD)

The inhibition of reduction of nitroblue tetrazolium (NBT) to blue colored formozan in presence of phenazine methosulphate (PMS) and Nicotinamide adenine dinucleotide (NADH) was measured at 560 nm using n-butanol as blank. Briefly, to 0.2 mL of tissue homogenate was added 0.6 mL of 0.052 M sodium pyrophosphate buffer (pH 8.3), 50 µL of 186 µM of PMS, 150 µL of 300 µM NBT, and 0.4 mL of distilled water. Reaction was started in the above by the addition of 0.1 mL of 780 µM NADH. After incubation at 30°C for 60 sec, the reaction was stopped by the addition of 0.5 mL of glacial acetic acid. The reaction mixture was stirred vigorously and shaken with 2 mL of n-butanol. The mixture was allowed to stand for 10 min, centrifuged at 3000 rpm for 10 min and butanol layer was taken out. Colour intensity of the chromogen in the butanol was measured at 560 nm in spectrophotometer against n-butanol, a system devoid of enzyme that served as control. One unit of enzyme activity is defined as enzyme concentration required inhibiting the optical density at 560 nm of chromogen protection by 50% in one min under the assay conditions. The results were expressed as IU/mg protein [10].

#### 2.7.3. Estimation of Catalase (CAT)

Tissue is homogenized (5%) in M/150 phosphate buffer at 1–4°C and centrifuge at 1000 rpm for 30 minutes. The supernatant so obtained was diluted 1 : 10 with water and 0.04 mL was taken for the assay. Decomposition of H_2_O_2_ in presence of catalase was followed at 240 nm. The results were expressed as nU/mg protein [11].

### 2.8. Estimation of Granulation Tissue Free Radicals in Wet Tissue

#### 2.8.1. Estimation of Lipid Peroxidation (LPO)

0.2 mL of 100 mg/mL tissue homogenate was added 0.1 mL of 8.1% SDS, 0.75 mL of 20% acetic acid solution (pH 3.5), and 0.75 mL of 0.8% aqueous solution of TBA in stoppered tubes. The mixture was made up to 2 mL with distilled water and then heated in an oil bath at 95°C for 60 minutes. After cooling with tap water, 0.5 mL of distilled water and 2.5 mL of mixture of n-butanol and pyridine (15 : 1, v/v) were added and shaken vigorously. After centrifugation at 3000 rpm for 10 min, the organic layer was taken and its absorbance at 532 nm was measured against blank containing 0.2 mL of distilled water in place of sample. The results were expressed as nM/mg protein [12].

#### 2.8.2. Estimation of Nitric Oxide (NO)

As nitrite and nitrate are formed as end products of the reactive nitrogen intermediates, the measurement of nitrite by using the Griess reagent is generally employed as a marker for formation of NO. 0.4 mL of granulation tissue homogenate (100 mg/mL) is mixed with 0.4 mL of absolute alcohol and then centrifuged at 4°C at 14000 rpm for 1 hr. 0.5 mL of supernatant was taken, mixed with 0.5 mL of vanadium (III) chloride and 0.5 mL of freshly prepared Griess reagent, and incubated at 37°C for 30 min. The absorbance was measured at 540 nm by spectrophotometer, against blank prepared by using distilled water. The results were expressed as nM/mg protein [13].

### 2.9. Estimation of Myeloperoxidase (MPO)

For MPO estimation, granulation tissue (5% w/v) was homogenized in 0.5% HTAB with 50 mM potassium phosphate buffer (pH 6). The above homogenate was freeze-thawed three times and sonicated for 10 seconds and then centrifuged at 14000 ×g for 45 minutes at 4°C and the resulting supernatant was used for estimation of MPO. A unit of MPO activity is defined as that converting 1 *μ*mol of H_2_O_2_ to water in 1 min at 25°C. The results were expressed as mU/mg protein [14].

### 2.10. Estimation of Connective Tissue Parameters

The dry granulation tissue (40 mg/mL) in 6 N HCl was kept in boiling water bath for 24 h for hydrolysate. The hydrolysate was cooled and excess of acid was neutrilised by 10 N NaOH using phenolphthalein indicator. The volume of neutral hydrolysate was diluted to a concentration of 20 mg/mL with distilled water. The final hydrolysate was used for estimation of hydroxyproline, hexuronic acid, and hexosamine following the standard procedure [4].

### 2.11. Estimation of Protein

0.05 mL of alcoholic precipitate of granulation tissue homogenate was dissolved in 1 mL of 0.1 N NaOH. 0.4 mL of the above sample was taken into another test tube and to which 4 mL of alkaline reagent was added and kept for 10 minutes. Then 0.4 mL of the phenol reagent was added and again 10 minutes were allowed for color development. Readings were taken against the blank prepared with water at 610 nm. The results were expressed as mg/g wet or dry tissue [15].

### 2.12. Histopathology

The cross-sectional full-thickness skin specimens and granulation tissues from the implanted tube were collected on 10th day of the experiment for the histopathological alterations. Samples were fixed in 10% buffered formalin, processed and blocked with paraffin, and then sectioned into 5 *μ*m sections and stained with hematoxylin and eosin.

### 2.13. Limit Test Study

Adult Swiss albino mice of either sex, weighing between 20 and 25 g fasted overnight, were used for toxicity study. Suspension of AME was administered orally at 2 g/kg stat dose (10 times of the optimal effective dose) to mice. Subsequent to extracts administration, animals were observed closely for first 4 h, for any toxicity manifestation, like increased motor activity, salivation, convulsion, coma, and death. Subsequently observations were made at regular intervals for 24 h. The animals were under further investigation up to a period of two weeks [16, 17].

### 2.14. Statistical Analysis

Statistical comparison was performed using either unpaired *t-*test or one-way analysis of variance (ANOVA) for multiple comparisons versus control group done by Dunnett's test. All statistical analysis was performed using SPSS statistical version 16.0 software package (SPSS Inc., USA).

## 3. Results

### 3.1. Incision Wound

Control rats showed WBS as 335.0 ± 15.9 g on 10th postwound day. AME extract 100, 200 and 400 mg/kg treated rats showed increase in WBS by 22.0, 44.4 and 38.8% (*P* < 0.01 to *P* < 0.001) respectively and the effect on WBS was comparable with VTE treated rats (57.9%, increase, *P* < 0.001) (Table 1).

### 3.2. Excision Wound

**Figure 1:**
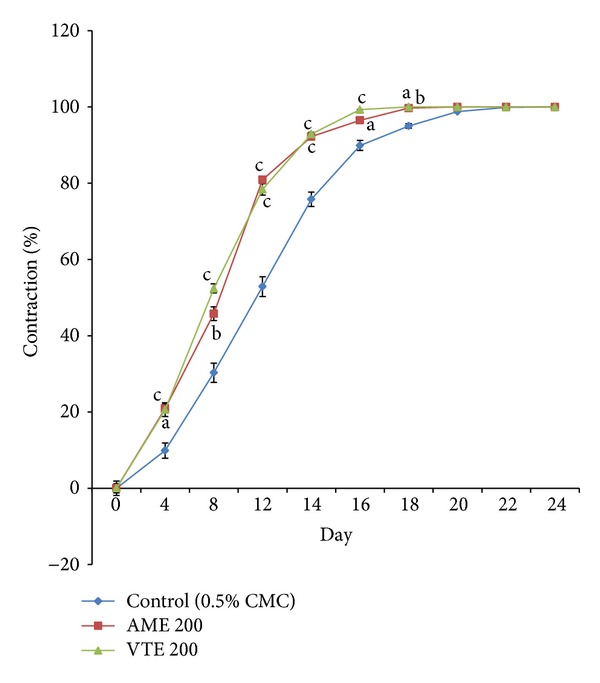
Effect of AME and VTE on percent wound contraction in excision wound. Range of 0 day wound area of control, AME, and VTE group (409.6 ± 17.9 to 418.0 ± 21.0 mm^2^). Results are mean ± SEM percent wound contraction from respective 0 day value (*n* = 6). ^a^ *P* < 0.05, ^b^ *P* < 0.01, and ^c^ *P* < 0.001 compared to respective day control group (statistical analysis was done by one-way analysis of variance followed by Dunnett's test for multiple comparisons).

**Figure 2:**
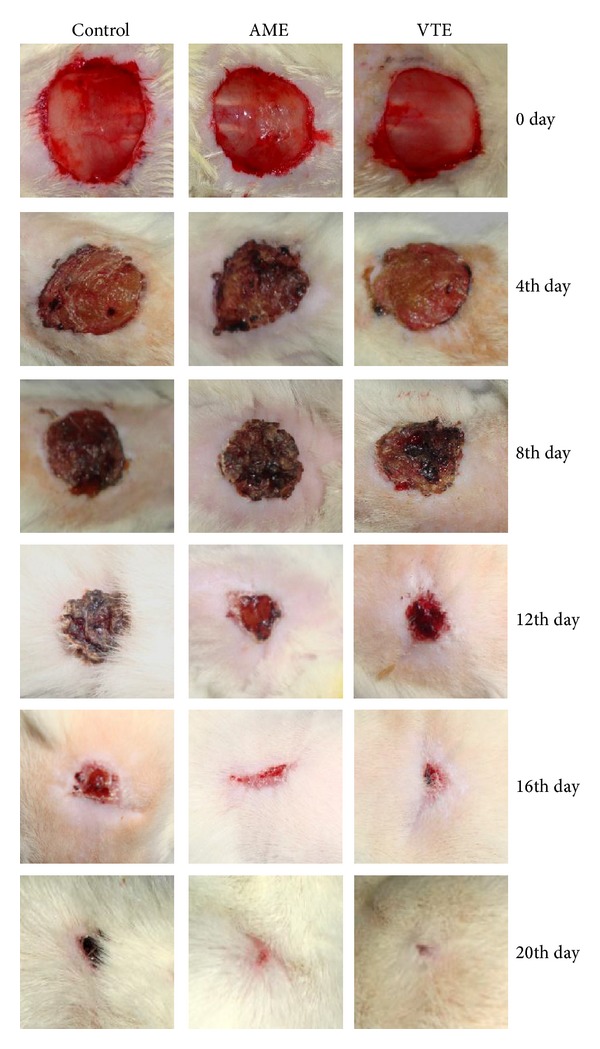
Photographic representation of percent wound contraction on different postexcision days of control, AME (200 mg/kg) and VTE (200 mg/kg) treated rats on day 4 (Control: 9.9, AME: 20.9, VTE: 20.6), day 8 (control: 30.3, AME: 45.8, VTE: 52.4), day 12 (control: 52.9, AME: 80.9, VTE: 78.4), day 16 (control: 89.9, AME: 96.5, VTE: 99.3), and day 20 (control: 98.8, AME: 100, VTE: 100).

Rate of wound contraction in control rats was 9.9% to 52.9% from day 4 to day 12 and 75.8% to 98.8% from day 14 to day 20, while complete normalization of skin was observed on day 24. Average number of days that took the shedding eschar without leaving any residual raw wound (epithelization period) was 15.3 days and mean scar area was 52.2 mm^2^. AME showed time-dependant healing effect on wound surface area in excision wound in rats. The percent rate of wound contraction in AME-treated rats was 20.9% on day 4 to 80.9% on day 12 and 92.2% to 99.7% from day 14 to day 18, respectively. VTE-treated rats showed increase in wound contraction from 20.6% on day 4 to 78.4% on day 12 and 92.9% to 100% from day 14 to day 18. The mean epithelization period and scar area were 13.5 and 13.2 days and 36.5 and 37.5 mm^2^ in AME and VTE treated groups, respectively (Figures 1 and 2).

**Figure 3:**
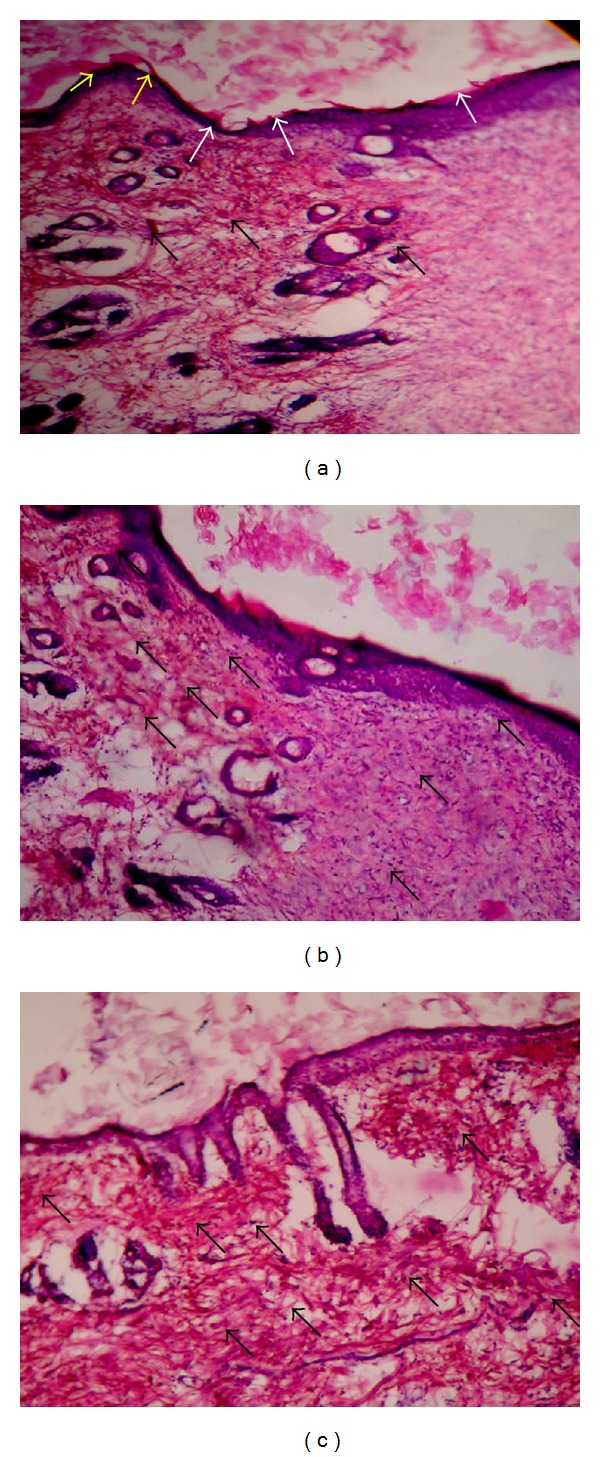
Histopathology of skin at day 10 stained with H&E (100x). (a) Skin of control rat showing ulceration and edema showed by white arrow, early epithelization showed by yellow arrow, and granulation tissue and abundance of mononuclear inflammatory cells showed by black arrow. (b) AME treated rats showing large amount of granulation tissue by black arrow, small number of mononuclear inflammatory cells, and restoration of adnexa and extensive fibrosis. (c) VTE treated rats showing healed skin structures with near to normal epidermis, restoration of adnexa and extensive fibrosis and collagen tissue within the dermis.

Histology of excision biopsy of skin wound at day 10 showed healed skin structures with normal epithelization, restoration of adnexa, and fibrosis within the dermis in AME and VTE treated groups while the control group lag behind treated group in formation of the amount of ground substance in the granulation tissue, as observed in tissue sections (Figure 3).

### 3.3. Dead Space Wound

#### 3.3.1. Granulation Tissue Weight and Protein Content

Control group showed wet granulation tissue weight as 237.8 ± 11.4 mg/100 g body weight and protein content as 67.8 ± 1.9 mg/g wet tissue. AME increased wet weight of granulation and protein by 46.3% (*P* < 0.001) and 8.4% (*P* < 0.05), respectively, compared with control group.

#### 3.3.2. Antioxidants, Free Radicals, and Myeloperoxidase

AME showed significant increase in the level of antioxidants, GSH, SOD, and CAT while free radicals, LPO and NO, and acute inflammatory marker; MPO was decreased (Table 2).

#### 3.3.3. Collagen Determinants. 

**Figure 4:**
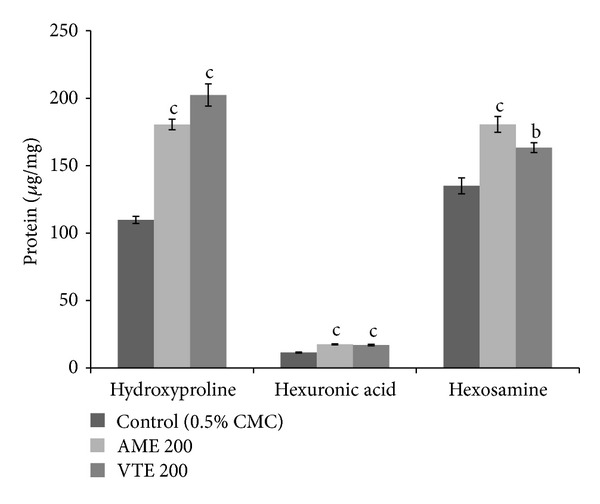
Effect of AME and VTE on dry granulation tissue hydroxyproline, hexosamine, and hexuronic acid content. Results are mean ± SEM (*n* = 6). ^a^ *P* < 0.05, ^b^ *P* < 0.01, and ^c^ *P* < 0.001 compared to respective control group (statistical analysis was done by one way analysis of variance followed by Dunnett's test for multiple comparisons).

Control group showed dry granulation tissue weight as 68.3 ± 4.10 mg/100 g body weight and protein content as 239.8 ± 15.3 mg/g dry tissue. Contents of both granulation tissue weight (18.9% increase, *P* < 0.05) and protein (15.2% increase, *P* < 0.05) were increased by AME treatment. Further, connective tissue parameters like hydroxyproline (control 109.8 ± 2.6 *μ*g/mg protein), hexuronic acid (control 11.4 ± 0.4 *μ*g/mg protein), and hexosamine (control 135.1 ± 5.9 *μ*g/mg protein) were significantly increased in AME treated group by 64.4% (*P* < 0.001), 53.8% (*P* < 0.001), and 33.7% (*P* < 0.01), respectively (Figure 4).

**Figure 5:**
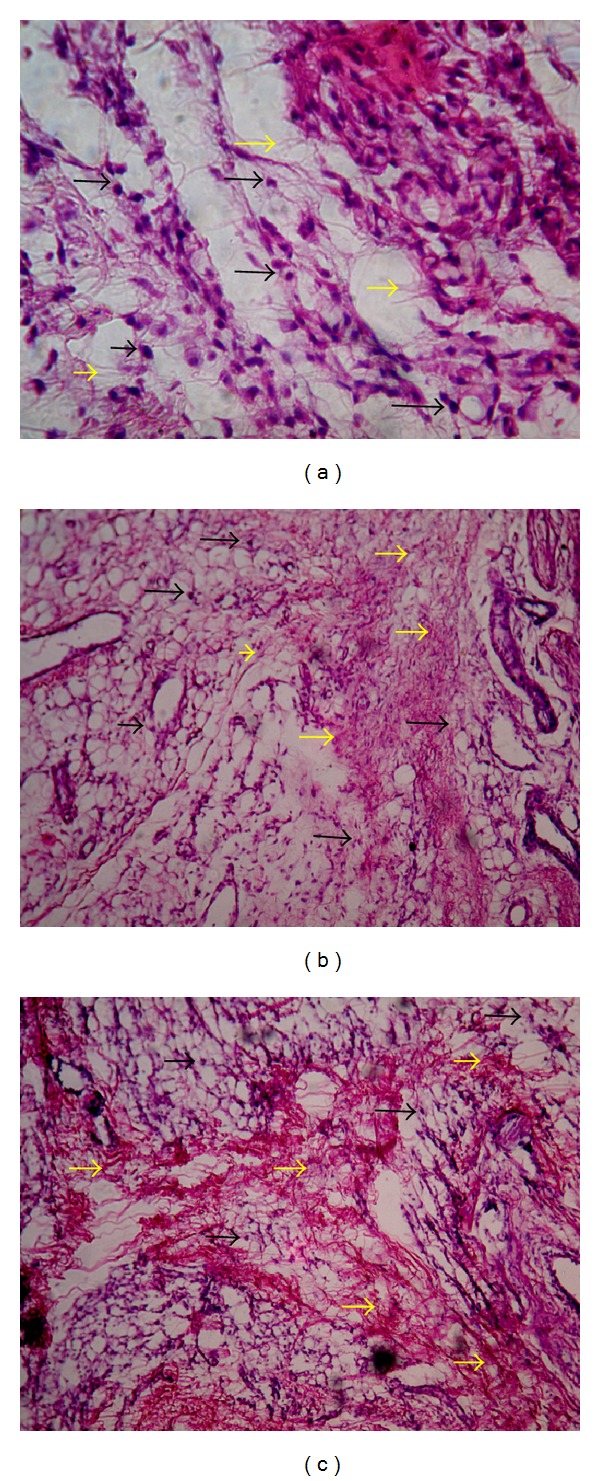
Histopathology of granulation tissue at day 10 stained with H&E (100x). (a) Granulation tissue of control rat showed mononuclear inflammatory cells (marked by black arrow), scattered abundance of eosinophilic fibroblasts (marked by yellow arrow). (b) AME treated showing large number of collagen tissue (fibrosis) and neovascularisation with minimal inflammatory cells. (c) VTE treated showing near to normal features, collagen tissue (fibrosis), and neovascularisation.

Histology of granulation tissue of control rat showed mononuclear inflammatory cells, scattered fibroblasts (minimal fibrosis), and few proliferating vasculature in granulation tissue, while the granulation tissue of rats treated with AME and VTE showed abundance of eosinophilic collagen tissue and neovascularisation with few inflammatory cells indicative of healing by fibrosis (Figure 5).

### 3.4. Limit Test Study

AME even at their ten times of optimal effective doses, that is, 2 g/kg, when given in stat dose, orally, did not show any acute toxic manifestation like increased motor activity, compulsive behavior, salivation, colonic convulsions, coma, or death, when observed up to a period of two weeks.

## 4. Discussion

Wound healing is a complicated process. It is a response to injury aimed at reconstructing damaged tissue and requires precise coordination of connective tissue repair, re-epithelialization, and angiogenesis. To generate new tissue and heal the wound, fibroblasts not only proliferate to increase cell numbers, but also produce several extracellular matrix proteins and growth factors [18]. A therapeutic agent selected for the treatment of wounds should ideally improve one or more phases of healing without producing deleterious side effects. Plant products are potential agents for wound healing and largely preferred because of their widespread availability, minimal toxicity, absence of unwanted side effects, and their effectiveness as crude preparations [19]. When tissue is first wounded, blood comes in contact with collagen, triggering blood platelets to begin secreting inflammatory factors. Platelets, the cells present in the highest numbers shortly after a wound occurs, release a number of things into the blood, including growth factors [3]. Platelets also release other proinflammatory factors to increase cell proliferation and migration of leucocytes. About two or three days after the wound occurs, fibroblasts begin to enter the wound site, marking the onset of the proliferative phase even before the inflammatory phase has ended [20]. In the present study, AME significantly enhanced the rate of wound contraction and epithelization period. Histology of excision wound also showed normal epithelization, adnexa, and fibrosis within the dermis. Wound contraction is the process of mobilizing healthy skin surrounding the wound to cover the denuded area and involves complex and superbly orchestrated interaction of cells, extracellular matrix, and cytokines. This centripetal movement of wound margin is believed to be due to the activity of myofibroblast. Since AME enhanced wound contraction, it would have either enhanced contractile property of myofibroblasts or increased the number of myofibroblasts recruited into the wound area. Granulation, collagen maturation, and scar formation are some of the many phases of wound healing which run concurrently, but independent of each other [21].

The wound breaking strength is determined by the rate of collagen synthesis and maturation process, wherein there is binding of collagen fibers through inter- and intra-molecular cross-linking [22]. AME was found to increase wound breaking strength in incision wound study which may indicate increased collagen content, cross-linking collagen, and maturation by AME. The granulation tissue of the wound is primarily composed of edema, fibloblast, collagen, and new blood vessels. The mesenchymal cells of the wound areadjust themselves into fibroblast and then begin migrating into the wound gap together with fibrin strands. Collagen is a principal component of connective tissue, plays a key role in the healing of wounds, and provides a structural framework for the regenerating tissue [23]. Collagen is a constituent of growing cell in healing tissues, which can be measured by monitoring the concentration of hydroxyproline. Thus, higher concentration of hydroxyproline indicates faster rate of wound healing. Biochemical analysis showed increased hydroxyproline content, which is a reflection of increased cellular proliferation and thereby increased collagen synthesis. Increased hexosamine, hydroxyproline, and hexuronic acid content reflects the stabilization of collagen molecules by enhancing electrostatic and ionic interactions. Collagen not only confers strength and integrity to the tissue matrix but also plays an important role in homeostasis and in epithelialization in the later stages of wound healing [2]. Furthermore, increase in dry tissue also indicates the presence of elevated protein level. In our present study, AME treatment showed significant increase in the levels of tissue weight, protein, hydroxyproline, hexosamine, and hexuronic acid in dry granulation tissue in comparison to control animals. It may be due to increase in collagen turnover or elevated protein level as observed in our study. Histology of granulation tissue of AME treated rat showed abundance of eosinophilic collagen tissue and neovascularisation with few inflammatory cells indicative of healing by fibrosis.

Reactive oxygen species (ROS) includes oxygen-derived radical as well as nonradical oxidants, which are often loosely termed “oxidants or free radicals,” are vital part of healing, and serve as cellular messengers that drive numerous biological pathways. These include beneficial pathways of wound healing for example; at optimum micromolar concentration, hydrogen peroxide can promote vascular endothelial growth factor (VEGF) expression in keratinocytes [24]. Optimum concentration of ROS is fruitful for body but at higher concentrations, induce severe tissue damage and even lead to neoplastic transformation. It has been reported earlier that topical application of compounds with free radical scavenging properties in patients has shown to improve wound healing significantly and protect tissues from oxidative damage [2]. The significant alteration in the antioxidant profile accompanied by the elevated levels of MDA, a marker of free radical damage or lipid peroxidation, may be attributed to impaired wound healing in immunocompromised rats. Overproduction of reactive oxygen species (ROS) results in oxidative stress, thereby causing cytotoxicity and delayed wound healing. Therefore, elimination of ROS could be an important strategy in healing of chronic wounds [1] and estimation of antioxidants like superoxide dismutase (SOD), catalase (CAT), and reduced glutathione (GSH) in granulation tissues becomes relevant as they hasten the process of wound healing by destroying the free radicals. MPO, a major indicative of the inflammatory activity in the granulation tissue, exists in neutrophils and reflects degree of neutrophils infiltration. Wound ulcer undergoes substantial oxidative stress by neutrophils derived oxidants and MPO activity, both of which contribute markedly to tissue damage during chronic wound inflammation [25]. Our studies on the SOD, CAT, GSH, LPO, NO, and MPO status in granulation tissue revealed that AME possessed significant antioxidant activity, reducing free radicals stress and MPO level and this could help to prevent oxidative damage and promote the healing processes.

AME is reported to contain important bioactive compounds such as carotenoids, phenolics, alkaloids, pectins, tannins, coumarins, flavonoids, and terpenoids. Flavonoids are most commonly known for their antioxidant activity [26]. Tannins have been reported to possess antioxidant, wound healing, and antimicrobial activities. Phenolic compounds are commonly known for their antioxidant, anti-inflammatory, and antimicrobial activities, while triterpenoids have been reported to show immunomodulatory property. Flavonoids are known to reduce lipid peroxidation not only by preventing or slowing the onset of cell necrosis but also by improving vascularity [6]. The healing effects of AME in wound healing could be due to the presence of above active principles.

## 5. Conclusion

Thus, the wound healing effects on 50% ethanol extract of *Aegle marmelos *fruit pulp seemed to be due to decreased free radical and myeloperoxidase generated tissue damage, promoting effects on antioxidant status, faster collagen deposition as evidenced by increase in collagen determinants and decrease in inflammation confirmed histopathologically.

## Figures and Tables

**Table 1:** Effect of graded doses of AME on wound breaking strength in incision wound.

Oral treatment (od × 10 days)	Wound breaking strength (g)	PIWBS
Control 0.5% CMC	335.0 ± 15.9	0.0
AME 100 mg/kg	408.8 ± 11.4^b^	22.0
AME 200 mg/kg	483.8 ± 9.2^c^	44.4
AME 400 mg/kg	465.0 ± 10.4^c^	38.8
VTE 200 mg/kg	528.8 ± 9.3^c^	57.9

Results are mean ± SEM (*n* = 6). Percentage increases in wound breaking strength (PIWBS) = (*D*_*T*_/*D*_*C*_ − 1) × 100, where *D*_*T*_ and *D*_*C*_ are WBS values of respective AME-treated and control group.

^b^ *P* < 0.01, ^c^ *P* < 0.001 compared to control group (statistical analysis was done by one-way analysis of variance followed by Dunnett's test for multiple comparisons).

**Table 2:** Effect of AME and VTE on free radicals (LPO & NO), antioxidants (SOD & GSH), and myeloperoxidase in granulation tissue.

Oral treatment (mg/kg, od)	Antioxidants			Free radicals		Myeloperoxidase
	GSH nM/mg protein	SOD IU/mg protein	CAT mU/mg protein	LPO nM/mg protein	NO nM/mg protein	MPO mU/mg protein
Control (0.5% CMC)	27.7 ± 0.98	0.43 ± 0.02	43.1 ± 1.69	5.82 ± 0.10	39.00 ± 1.19	23.9 ± 0.43
AME 200	31.3 ± 0.76^a^	0.67 ± 0.05^b^	180.2 ± 0.67^c^	2.62 ± 0.16^c^	17.34 ± 0.63^c^	18.8 ± 0.42^c^
VTE 200	36.3 ± 1.12^c^	0.81 ± 0.03^c^	211.7 ± 0.95^c^	2.33 ± 0.10^c^	17.75 ± 0.70^c^	15.1 ± 0.58^c^

Results are mean ± SEM (*n* = 6). ^a^ *P* < 0.05, ^b^ *P* < 0.01, and ^c^ *P* < 0.001 compared to respective control group (statistical analysis was done by one way analysis of variance followed by Dunnett's test for multiple comparisons).
